# Bridging Hypoxia and Vision Loss: The Emerging Role of Connexins in Local and Systemic Eye Diseases

**DOI:** 10.3390/ijms27020886

**Published:** 2026-01-15

**Authors:** Xianping Zhang, Yalong Cheng, Jean X. Jiang, Yuting Li

**Affiliations:** 1 Department of Pathology, Basic Medical School, Ningxia Medical University, Yinchuan 750004, China; 2 Epidemiology and Health Statistics, Public Health School, Ningxia Medical University, Yinchuan 750004, China; 3 Department of Biochemistry and Structural Biology, University of Texas Health Science Center at San Antonio, San Antonio, TX 78229, USA

**Keywords:** hypoxia, connexin, gap junctions, hemichannels

## Abstract

Hypoxic eye diseases represent a pivotal yet often underappreciated contributor to the onset and progression of many retinal disorders. When hypoxia persists or exceeds the tissue’s compensatory capacity, it triggers pathological retinal neovascularization, blood–retinal barrier disruption, and neuronal apoptosis, ultimately resulting in irreversible visual impairment. Connexins (Cxs) form gap junction channels and hemichannels and regulate retinal cell proliferation, differentiation, and survival, thereby playing a central regulatory role in the pathogenesis of hypoxic ocular diseases. In addition to gap junctions, Cx hemichannels promote transmission of molecules between intra- and extracellular environments, further influencing retinal homeostasis under hypoxic stress. This review synthesizes recent progress in understanding connexins in localized and systemic hypoxic eye diseases. We focus on the molecular mechanisms underlying the development and progression of hypoxia-induced ocular pathology, with particular emphasis on the emerging potential of Cxs as novel therapeutic targets for hypoxic ocular diseases. Following a systematic literature search, the electronic databases PubMed and EMBASE were consulted, with the search deadline set at December 2025. The search terms employed were as follows: hypoxia, connexin, gap junctions, hemichannels.

## 1. Introduction

As an organ that is dependent on oxygen supply to maintain its fundamental functions, the eye exhibits significantly greater sensitivity to hypoxia than other tissues [1]. The metabolically active retinal neuroepithelium, optic nerve, and choroidal vascular cannot tolerate pressure of oxygen [2]. During the pathological progression of hypoxic ophthalmopathy, local hypoxia initially disrupts the balance between proliferation and apoptosis in retinal vascular endothelial cells (RECs), leading to disorganized luminal structures [3]. Abnormal dissociation of intercellular junctions further exacerbates the destabilization of the tissue microenvironment. Retinal ganglion cells (RGCs), central to transmitting visual signals to the brain, are most vulnerable to hypoxia [4]. Following hypoxia, mitochondria can generate substantial amounts of reactive oxygen species (ROS). Early mitochondrial dysfunction occurs, and as hypoxia persists, multiple pathological pathways are activated [5]. These include inflammatory cascades, abnormal ion channel activation, and amplified oxidative stress, ultimately leading to cell apoptosis and irreversible vision loss.

The damaging effects of hypoxia-associated ophthalmic disorders are well-established, with hypoxia identified as the core driver of pathological progression. However, current clinical treatments face significant challenges: anti-vascular endothelial growth factor (anti-VEGF) agents only selectively improve vascular proliferation [6]. The anti-inflammatory effects of drug carry side effects associated with long-term use. And destructive therapies like laser photocoagulation sacrifice partial retinal function [7]. Therapeutic approaches face the dilemma of clear pathological mechanisms but limited intervention targets, making it imperative to identify core regulatory factors of hypoxic eye diseases at the molecular level. Bridging the translational gap between pathological mechanisms and therapeutic targets has emerged as a novel research challenge.

Connexins (Cxs), as core molecules forming intercellular communication channels, elucidate their pathological mechanisms under hypoxic conditions while offering novel research avenues for addressing therapeutic challenges [8]. Research has confirmed that selective intervention targeting HC function significantly mitigates hypoxia-induced RGC damage, establishing them as novel therapeutic targets [9].

This review focuses on describing the expression characteristics of Cx family members in different ocular cells and the pathological mechanisms of hypoxia, exploring the molecular mechanisms through which they participate in the pathological process of hypoxic eye diseases. It explores intervention strategies and translational applications targeting Cx channels, aiming to fill scientific gaps in this field and provide theoretical foundations for novel therapeutic approaches.

### 1.1. Hypoxic Eye Diseases

As the vital organ for perceiving and processing visual signals, the eye is particularly vulnerable to hypoxic stress. It relies on its blood supply system to sustain its metabolic activity [10]. Ocular perfusion primarily depends on the internal and external carotid artery systems [11]. The central retinal vascular system predominantly covers the five layers within the retina, supplying oxygen and nutrients to retinal ganglion cells to support visual signal transmission [12]. The peripheral vascular system, meanwhile, serves the choroid, ciliary body, and iris [13]. Systemic hypoxia induces optic nerve axonal apoptosis, leading to progressive visual field constriction [14]. It also causes damage to RECs, resulting in luminal narrowing or occlusion [15]. Localized hypoxia, such as that associated with age-related macular degeneration (AMD), impairs substance exchange between the retinal pigment epithelium (RPE) and choroidal capillaries, ultimately causing choroidal vascular atrophy [16]. The eye depends on specific arterial perfusion systems and is vulnerable to hypoxic stress, which causes various ocular damages.

### 1.2. Cxs in Eye Tissues

Cxs are important for maintaining ocular homeostasis and visual function. In humans, the family of connexins, or GJ proteins, contains 21 members with shared structural features [17]. In the lens, Cxs are pivotal for epithelial wound healing and barrier function. Studies have shown that the len epithelium predominantly expresses Cx43 and Cx50 under quiescent conditions, which facilitate metabolic coupling and intercellular communication to preserve epithelial integrity [18]. Retinal neurons and glial cells express multiple Cx isoforms, including Cx36, Cx43, and Cx45 [19]. Cx36 is primarily localized in the inner and outer plexiform layers, and this distribution enables the rapid transmission and integration of visual signals, critical for visual acuity [20]. Cx43, expressed in Müller and RPE, supports nutrient transport and waste removal. In the ciliary epithelium, which secretes aqueous humor to maintain intraocular pressure (IOP), Cxs facilitate the transport of ions and small molecules required for aqueous humor production. In the optic nerve, recent research has identified novel roles of Cxs in maintaining optic nerve function. GJs formed by Cx43 create electrical synapses between axons in the optic nerve head. These synapses enable lateral signal conduction, which may accelerate the transmission of visual signals from the retina to the brain and help bypass local axonal damage. Cxs are critical for ocular homeostasis and vision.

### 1.3. Structure and Functions of Connexins, Gap Junctions and Hemichannels

The family of connexins, or GJ proteins, contains 21 members in human with shared structural features [21]. Each Cx comprises four transmembrane α-helical domains linked by two extracellular loops, which are crucial for intercellular recognition and docking [22]. Cxs possess cytoplasmic domains at both the N- and C-termini, with the cytoplasmic ring linking the second and third transmembrane domains in co-connexins [23]. Electron crystallography reveals each HC contains six Cxs monomers arranged in a hexagonal structure [24]. The docking of two Cxs, also known as connexon, from adjacent cells bridges the extracellular space, creating a GJ channel [25,26]. The small projections of the extracellular domains of the paired Cxs results in a 2-3nm separation (gap) between the junctional membrane [27]. Gap junctions can protect astrocytes by permitting the exit of toxic molecules, including Ca2+, excessive ATP, Glu, NO, and ROS, out of the injured astrocyte and the entrance of neuroprotective metabolites [28]. The function of the HCs promotes the maintenance of blood-retinal barrier (BRB) integrity associated with GJ closure under normal conditions [29]. Heterogeneous GJ channels exhibit distinct unit conductance and gating properties compared to homogeneous channels [30]. In Xenopus oocytes oocytes co-expressing Cx40 and Cx43, GJICs demonstrate divergent pH gating properties, illustrating the influence of subunit combinations on gating functionality [31]. Transmembrane selectivity exists within the active processes of GJ, that canonically physiological functions [32]. Cxs ensures precise electrical signaling in maintaining retina [33], liver [34], cardiac homeostasis [35]. GJ is critically significant in maintaining tissue function and intercellular communication.

### 1.4. The Relationship Between Cxs and Hypoxic Eye Diseases

**Figure 1:**
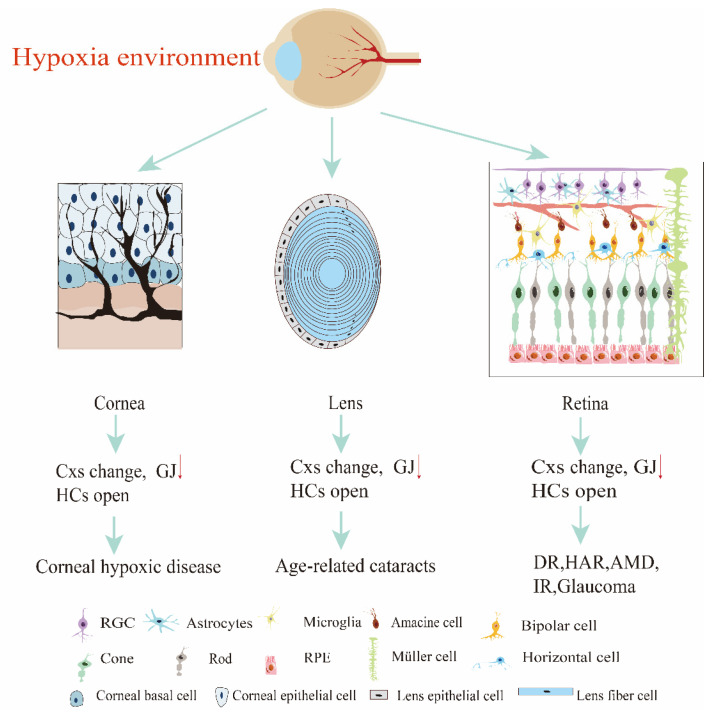
The eye was damaged under a hypoxic environment. Cx: connexin; GJ: gap junction; HC: hemichannel; DR: diabetic retinopathy; HAR: high-altitude retinopathy; AMD: age-related macular degeneration; IR: ischemic retinopathy; RGC: retinal ganglion cell; RPE: retinal pigment epithelium.

Cxs play an indispensable role in maintaining retinal microenvironmental homeostasis. In inflammatory states, Cx43 protein expression is closely related to microglia activation, loss of astrocytes, and the release of inflammatory factors [36]. Researchers have confirmed that mild to moderate injury leads to increased Cx43 immunoreactivity at lesion sites [24,37], whereas severe injury results in decreased Cx43 immunoreactivity in injured areas [38]. Dynamic changes in the retinal Cx43 protein are associated with RGC and REC loss and inflammatory responses [39,40]. We focus particularly on those associated with hypoxia, encompassing systemic hypoxia-induced ocular pathologies, including diabetic retinopathy (DR), high-altitude retinopathy (HAR), ischemic retinopathy (IR), hypertension, diabetic glaucoma, and metabolic cataracts, as well as local hypoxia-induced ocular diseases, including AMD, corneal hypoxic disease, glaucoma, and age-related cataracts. Retinal hypoxia stimulus initiates neovascularization in ocular pathologies, such as DR, AMD, and vascular occlusions (Table 1). Cx is implicated in numerous ophthalmic disorders (Figure 1).

## 2. Cxs, GJs, and HCs in Hypoxic Retinal Diseases

### 2.1. Role of Hypoxia in Diabetic Retinopathy

Diabetic retinopathy (DR) is a common and specific microvascular complication of diabetes mellitus and is the primary cause of blindness [47]. Clinical manifestations are divided into two stages: non-proliferative diabetic retinopathy (NPDR) and proliferative diabetic retinopathy (PDR) [48,49]. Retinal arterial dilation may serve as an early physiological marker of hypoxia-induced microvascular dysfunction, with its core mechanism closely linked to impaired arterial autoregulation under hypoxic conditions [50]. Within a hypoxic microenvironment, reduced tissue oxygen partial pressure in the retina triggers the dysfunction of vascular smooth muscle cells [50,51]. Persistent hypoxic stress and pressure overload further compromise capillary endothelial cell integrity. On the one hand, disruption of GJs increases vascular permeability, causing vascular leakage that forms retinal edema and hard exudates; on the other hand, hypoxia-induced oxidative stress exacerbates collagen fiber degradation in the vascular wall, thinning capillary walls and increasing their fragility, ultimately precipitating microaneurysm formation and vascular rupture with hemorrhage [52]. Considering these mechanisms collectively, retinal arteriolar dilation represents an early subclinical signal of hypoxia-mediated microvascular dysfunction, with its pathological progression preceding the emergence of typical clinical symptoms characteristic of non-proliferative diabetic retinopathy [53].

### 2.2. Connexin Expression Changes (Subtype and Cell Types)

Hyperglycemia (HG) damages RECs, increases blood viscosity, and induces vascular occlusion, directly causing local retinal hypoxia [41]. As a central pathological link in DR progression, hypoxia and HG synergistically regulate the expression of Cx43 in retinal microvascular endothelium. Abnormal Cx43 expression subsequently compromises BRB integrity, ultimately driving DR development [54]. The BRB comprises an inner barrier (RECs and their tight junctions) and an outer barrier (RPE cells and their tight junctions), whose integrity relies on the stable expression and functional integrity of tight junction proteins [55]. Diminished expression of tight junction proteins directly disrupts the structural connections between RECs and RPE cells, increasing the permeability of the cell monolayer and impairing the barrier function of the BRB [56]. This decreased expression of tight junction proteins is accompanied by increased permeability of the cell monolayer, indicating impaired BRB function [41]. Hypoxia research by Dongjoon Kim et al. showed that in the early stages of DR and under mild hypoxia, pathological HG and inflammatory stimuli increase Cx43 expression in RPEs, accompanied by a reduction in REC numbers [57,58,59], suggesting that early Cx43 upregulation may accelerate pathological cell death by disrupting intercellular communication. As DR progresses and hypoxia intensifies, Cx43 expression exhibits a marked downward trend, occurring extensively across multiple key retinal cell types. Studies confirm reduced Cx43 expression levels in RECs, peripapillary cells, Müller cells, RPEs, and RGCs in both DR patients and HG-induced DR models [41]. Research by Michael W Bobbie et al. further established the characteristic marked reduction in Cx43 expression throughout the DR disease course [33]. Persistent downregulation of Cx43 directly disrupts intercellular signaling and functional coordination within the retina, impairing retinal blood flow regulation mechanisms and exacerbating hypoxic conditions [54]. Collectively, Cx43 expression exhibits a dynamic pattern of early adaptive upregulation followed by late pathological downregulation in DR pathogenesis: during early DR, mild hypoxia induced by HG and inflammation may initiate cellular stress responses via Cx43 upregulation. As the disease progresses, persistent and worsening hypoxia induces a marked downregulation of Cx43 expression. This disrupts intercellular communication and the tight junction structure of the BRB, thereby exacerbating retinal ischemia–hypoxia and barrier damage.

### 2.3. GJ vs. HC Mechanisms

Under hypoxic conditions, including tissue injury, inflammation, and pathological states, HCs become hyperactivated. Microvascular injury and the concomitant loss of RECs and pericytes are associated with alterations in GJ and reduced Cx expression [60]. In hypoxia-mediated retinal injury and the pathological progression of DR, the hypoxia-induced opening of HCs is recognized as a pivotal step in secondary damage. This process not only induces glial cell activation, retinal edema, and loss of vascular integrity, but also ultimately accelerates retinal neuronal death [61]. Transient HC opening and increased intracellular Ca^2+^ in vitro can simulate the reduction in cellular viability induced by an ischemic environment [62]. Research indicates that the expression and activity of HCs are upregulated in DR, becoming a key factor in establishing the pro-inflammatory state that emerges during HG periods [63].

### 2.4. Role of Hypoxia in Age-Related Macular Degeneration

Age-related macular degeneration (AMD) is a chronic, progressive retinal degenerative disease [64] characterized by damage to the RPE and macular dysfunction that ultimately leads to central vision loss [65,66]. Oxidative stress and hypoxia in the RPE have long been recognized as core contributors to disease initiation and progression in AMD pathology [67]. Hypoxia within the retinal microenvironment arises from multiple pathological processes in AMD, including choroidal hypoperfusion, drusen-induced impairment of oxygen diffusion to the RPE, and metabolic dysfunction of retinal cells [68]. Patients may experience symptoms including blurred vision, headaches, visual distortions, reduced vision in one or both eyes, difficulty adapting to low-light environments, and an increased need for higher contrast sensitivity [69].

### 2.5. Connexin Expression Changes (Subtype and Cell Types)

The research team led by Huang Guohui et al. observed Cx43 expression levels increased in AMD [70]. In control retinas, Cx43 exhibits uniform distribution within both RNFL and RGC. Its expression diffusely extends towards the inner plexiform layer and inner nuclear layer [71]. In choroidal neovascularization, retinal cells predominantly express Cx37, Cx40, and Cx43, which effect REC proliferation, migration, and tubular structure formation [72]. Under hypoxic conditions, RECs upregulate Cx37 and Cx43 expression, promoting pathological angiogenesis [73]. Cx43 expression is upregulated via hypoxia-inducible fastrocytetor-1α (HIF-1α) binding to hypoxia response elements (HREs) [74]. Functional studies indicate that increased Cx43 expression enhances gap junctional information communication (GJIC), facilitating the transmission of pro-angiogenic signals, such as vascular endothelial growth factor (VEGF), between adjacent endothelial cells [75]. This enhanced intercellular communication promotes REC proliferation, migration, and tubule formation. In hypoxic conditions, the production of reactive oxygen species (ROS) in the endothelial cells stimulated with VEGF ultimately induces REC apoptosis [76].

### 2.6. GJ vs. HC Mechanisms

Overexpression of Cxs in AMD causes premature opening of HCs in the cell membrane. Marijke De Bock et al. investigated intact brain capillary endothelial cells isolated from LPS-treated mice, the HCs of which are termed ‘pathological pores’ [77]. Alice McDouall et al. found that, triggered by ATP release through these pathological pores, ATP enters the extracellular space and acts as an inflammasome signal 2 activator in Perinatal Brain Injury [78].

### 2.7. Role of Hypoxia in High-Altitude Retinopathy

High-altitude retinopathy (HAR) is a retinal disorder caused by prolonged exposure to hypoxic environments at high altitudes, which leads to severe visual impairment [79]. HAR typically does not impair vision, or causes only mild vision loss and slight visual field defects [80]. In such cases, the fundus examination usually reveals only retinal vascular dilation and tortuosity, cotton-wool spots, and minimal retinal hemorrhages not involving the macular area [81]. Rare but severely vision-impairing cases include ischemic optic neuropathy, cortical amaurosis, and permanent myopia following high-altitude exposure (HAE) [82]. Visual contrast sensitivity significantly increases within 15 min after brief exposure to low-pressure hypoxia [83]. Not all of these symptoms necessarily occur, and their manifestation varies with altitude, ascent rate, individual susceptibility, and other environmental factors [84]. Previous research has found that 25.9% of study participants exhibited vascular dilation and tortuosity in hypoxic environments. Other studies have reported that dilated and tortuous blood vessels contribute to the development of HAR [85].

### 2.8. Connexin Expression Changes (Subtype and Cell Types)

Hypoxia mediates two core pathological processes—BRB disruption and neuronal cell death—by regulating Cx expression levels across distinct retinal cell types [86,87,88,89,90]. Concurrently, elevated Cx expression in retinal ganglion cells mediates secondary cell death in HAR models [91,92]. Our team’s research further found that during acute HAE, Cx43 immunoreactivity in retinal tissue markedly intensified. This finding aligns strongly with the severe neural damage indicated by substantial RGC loss 72 hours post-HAE [88]. We observed inflammatory activation of microglia under HAE conditions, with retinal IBA1 protein showing the expression of microglia [43]. Our research reveals that hypoxia in the HAR model significantly upregulates Cx43 expression in RECs. Upregulation of cx43 expression disrupts synergistic regulation among RECs, leading to increased barrier permeability and ultimately BRB damage and retinal edema (unpublished). This suggests that Cx43 may participate in mediating the toxic effects of microglia activation on the RECs and RGCs by regulating the neuroinflammatory microenvironment, ultimately leading to substantial loss of RGCs. Cx43 may thus represent a pivotal target linking hypoxia, inflammation, and cellular injury within the pathological progression of HAE.

### 2.9. 2GJ vs. HC Mechanisms

Pía C Burboa et al. studied how bystander cell death affects small arteries through gap junction channels, and functional studies demonstrated a substantial increase in coupled astrocyte networks during the hypoxic phase [93]. This increase in GJ-mediated coupling was particularly evident in astrocytes located in the central avascular retina and occurred prior to their degeneration [94]. Preventing this increase in GJ coupling using HC inhibitors increased astrocyte density and promoted vascular remodeling. Research into the pathogenesis of HAR and the role of Cx43 continues to advance. Our team also hopes to protect RGCs by blocking functional HCs, thereby injecting new momentum into HAR treatment.

### 2.10. Role of Hypoxia in Ischemic Retinopathy

Ischemic retinopathy (IR) is a leading cause of irreversible vision loss globally. It is a disease driven by complex microvascular dysfunction, neuroinflammation, and tissue hypoxia [95]. IR results from insufficient retinal blood supply, causing hypoxia-induced pathological changes and potential vision impairment. The condition commences with impaired normal retinal blood supply, subsequently exacerbated by retinal neovascularization. Ischemia and the resulting oxidative stress render the already compromised retina more susceptible to oxidative damage [96]. Retinal ischemia is a key pathogenic factor in ocular diseases including ROP and DR, being closely associated with hypoxia in retinal tissue [91]. In the ischemic stroke model, functional disruption within central nervous system cells is repaired by increasing the local expression of GJ proteins in damaged neurons and glial cells [96].

### 2.11. Connexin Expression Changes (Subtype and Cell Types)

Elevated Cx expression enhances the formation of GJ between glial cells or between glial cells and neurons. However, sustained overexpression of these Cx channels exacerbates the transmission of pro-inflammatory signals and oxidative stress between retinal cells, ultimately leading to retinal neuronal apoptosis and promoting pathological neovascularization driven by HIF1α and VEGF [97]. Research indicates that hypoxia induced by retinal ischemia downregulates specific Cx isoforms within RECs in DR models. This reduction in Cx expression disrupts intercellular communication between RECs, a process critical for maintaining the integrity of the BRB [98]. The disruption of GJ structures leads to BRB dysfunction, manifesting as increased vascular permeability and the infiltration of plasma components into the retina [99].

### 2.12. GJ vs. HC Mechanisms

Research findings on diverse disease models indicate that Cxs and their mediated GJ functions assume a dual role as both ‘homeostatic maintainers’ and ‘amplifiers of injury’ in cerebral IR and traumatic disorders. In studies of brain tissue injury models, following initial damage triggered by stroke onset or epileptiform astrocytes, a core feature of this activation is enhanced GJ coupling between astrocytes [100].

The abnormal enhancement of GJ coupling not only accelerates the transmission of injury signals within the glial network but also amplifies the local tissue damage effects. This finding suggests that targeting the regulation of GJ function in reactive astrocytes may represent a potential therapeutic target for delaying the progression of brain injury.

Similarly to brain tissue, Cx function in retinal tissue exhibits marked alterations in ischemic injury models. As HCs serve as the single-channel structure for Cx assembly, their excessive activation acts as a conduit for the release of cytotoxic mediators, directly inducing secondary cell death in surrounding cells [92]. This establishes a vicious cycle where ischemic release of toxic mediators leads to further cell death. In models of IR, the avascular central retinal region exhibits markedly enhanced GJ coupling in adenosine cyclase-expressing astrocytes. The GJ-mediated coupling enhancement was particularly pronounced in neurons within the central avascular retinal region, followed by the degeneration of these neurons [101]. GJ coupling is not a passive consequence of cellular injury but rather an early, critical driver of retinal ischemic damage progression. This Cx dysfunction, occurring within specific cell populations, provides a clear molecular target and cellular vehicle for precise therapeutic intervention.

## 3. Ocular Diseases Resulting from Localized Hypoxia

### 3.1. Role of Hypoxia in Glaucoma

Glaucoma is a neurodegenerative disorder characterized by progressive degeneration of RGCs and their axons, ultimately leading to irreversible vision loss [102]. The primary mechanism by which prolonged elevated intraocular pressure damages the optic nerve is mechanical compression and impaired blood flow, leading to ischemia and hypoxia of the optic nerve fibers. During hypoxia, neurons are particularly susceptible to mitochondrial alterations, which lead to increased production of ROS [103].

#### 3.1.1. Connexin Expression Changes (Subtype and Cell Types)

Khulan Batsuuri et al. observed increased Cx43 expression in RGCs following the use of a mouse model with high intraocular pressure [44]. Kerr et al. discovered that elevated IOP significantly increased Cx43 expression, consistent with prior studies demonstrating increased Cx43 expression in the retinas and optic nerve heads (ONHs) of human glaucoma patients [104]. Cooper et al. demonstrated increased astrocytic coupling one week after IOP elevation following microsphere injection [105]. Another study found increased astrocytic Cx43 expression in retinal tissue from glaucoma mice and human glaucoma patients [37]. Astrocytes in glaucomatous eyes have noxious effects on optic nerve axons [106]. Elevated cx levels correlate with reactive astrocytic proliferation, revealing the harmful role of increased Cx43 in glaucoma progression. Research indicates that the hypoxic microenvironment in glaucoma significantly suppresses Cx36 expression levels in RGCs [107]. Reduced Cx36 expression directly disrupts signal transmission and functional coordination among RGCs. This subsequently leads to increased expression and abnormal distribution of other Cx proteins, ultimately causing damage to the retinal neuro-epithelial barrier and disruption of the retinal microenvironment [108].

#### 3.1.2. GJ vs. HC Mechanisms

In glaucoma, heightened HC activity may exacerbate glial cell hyperactivation, promoting the release of pro-inflammatory cytokines that further contribute to neuronal injury and optic nerve degeneration [109]. In the P2X7R-dependent signaling pathway, overproduction of ATP activates NLRP3 and inflammatory responses [110].

### 3.2. Role of Hypoxia in Retinopathy of Prematurity

Retinopathy of Prematurity (ROP) is an ocular disorder with a relatively high incidence among premature infants [111]. Its core pathological mechanism is closely associated with retinal hypoxic–ischemic states. The onset of retinal ischemia–hypoxia primarily stems from direct damage inflicted upon RECs by dramatic fluctuations in oxygen partial pressure during the immature development of a premature infant′s retinal vasculature. This damage triggers vascular occlusion, furthering exacerbates the vicious cycle of local ischemia–hypoxia [112], and this pathological process is closely associated with the abnormal expression of GJs within retinal tissue.

#### 3.2.1. Connexin Expression Changes (Subtype and Cell Types)

The pathological progression of ROP exhibits distinct stage specificity, divisible into hyperoxic and hypoxic phases [113]. The process of ROP includes retinal microvascular degeneration-associated vascularization in the peripheral retina [113]. The dysregulation of Cx expression is primarily concentrated during the hypoxic phase and is closely associated with VEGF-mediated pathological angiogenesis occurring in this stage [114]. This provides a theoretical basis for developing ROP treatment strategies targeting Cx-related pathways.

#### 3.2.2. GJ vs. HC Mechanisms

During the hypoxic phase of ROP, the toxic signals from dying cells are transmitted to healthy neighboring cells via gap junctions. This study demonstrates that Cx43 plays an irreplaceable role in astrocyte apoptosis preceding retinal neovascularization in an oxygen-induced mouse model of retinal disease [115].

### 3.3. Role of Hypoxia in Corneal Hypoxic Disease

The etiology of corneal hypoxia primarily arises from the cornea not being exposed to the atmosphere—such as during prolonged lens wear or prolonged eyelid closure [116]. Hypoxia also occurs in corneal inflammation and trauma when elevated oxygen demand exceeds supply [117]. Systemic hypoxia during pulmonary disease or HAE may similarly cause corneal hypoxia when the secondary oxygen consumption pathway via aqueous humor is compromised [118]. Hypoxia-related alterations in corneal structure and function include disturbances in corneal epithelial homeostasis, impairment of the corneal epithelial barrier function, reduced defensive capabilities, stromal edema, corneal congestion, and neovascularization [119]. The cornea has also evolved mechanisms to adapt to hypoxia induced by HIF [120].

#### 3.3.1. Role of Hypoxia in Connexin Expression Changes (Subtype and Cell Types)

In the stationary epithelium, Cx43 was localized exclusively in the basal cells [121]. Cxs exhibit differential distribution within tissues rather than being merely redundant [122]. Rabbit [123], mouse [124], and human [125] corneal epithelia simultaneously express both Cx43 and Cx50. Cx43 is consistently confined to basal cells, with no detectable presence in superficial cells [126]. Finally, the presence of Cxs in corneal endothelial cells (CECs) also appears to inhibit their proliferation [45].

#### 3.3.2. GJ and HC Mechanisms

One of the hallmark phenotypic features of the corneal epithelium is the formation of syncytial structures via GJs, which provide critical support for electrical signal transmission between epithelial cells [127]. As a GJ polypeptide initially identified in rodent lens fibers, Cx50 has been demonstrated to be equally present in the corneal epithelium [128]. The expression regulation of Cx43 is closely associated with corneal injury repair; knocking down Cx43 suppresses stromal edema and inflammatory responses [129]. This amelioration of the pathological microenvironment directly accelerates corneal endothelial injury healing and promotes epithelial re-epithelialization [24]. Gap junction-associated hemichannels maintain a closed state in normal. GJs participate in regulating corneal hypoxic diseases by maintaining stable communication between epithelial cells. When hypoxia occurs, the structure and function of GJs are disrupted, causing them to lose their supportive role in epithelial communication. This mechanism directly contributes to the onset and progression of corneal hypoxic diseases.

### 3.4. Role of Hypoxia in Age-Related Cataracts

Age-related cataracts (ARCs) are defined as the progressive clouding of the eye’s natural lens, constituting a primary cause of visual impairment and blindness [130]. Lens epithelial cells (LECs) exhibit significant upregulation of the glycolytic pathway when exposed to hypoxic conditions. It induces endoplasmic reticulum stress and ROS production within LECs, ultimately leading to apoptosis. Coupled with cumulative oxidative damage to the LECs, this leads to thickening and densification of the lens.

#### 3.4.1. Connexin Expression Changes (Subtype and Cell Types)

Cx50 is significantly downregulated in ARC lens cells. The expression of Cxs also undergoes alterations during aging, suggesting that the age-related loss of Cx50 contributes to the development of senile cataracts [131]. This indicates that one pathway through which hyperhomocysteinemia promotes cataract development is reduced Cx expression [132]. It has been discovered that mutations in the Cx46 and Cx50 caused cataracts in mice, with high-density mineral and calcium deposits visible in the mutated lenses [46]. Cxs regulate age-related cataracts by maintaining lens cell communication, and Cx dysfunction disrupts lens homeostasis, promoting protein aggregation and opacity.

#### 3.4.2. GJ and HC Mechanisms

**Figure 2:**
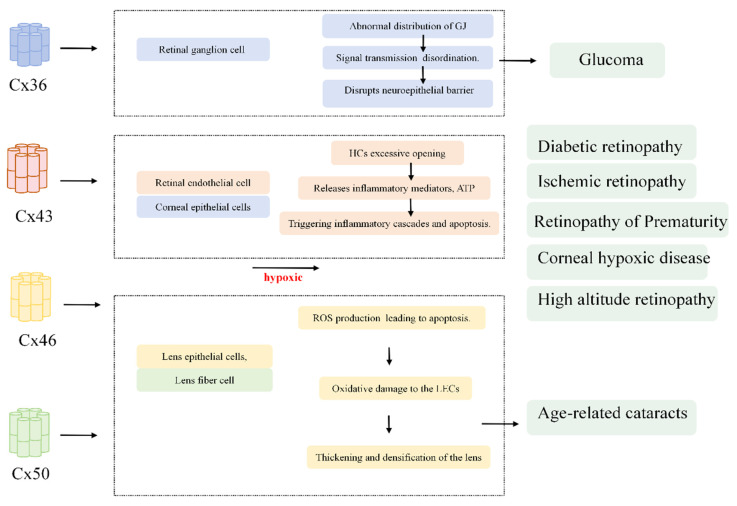
Hypoxia modulates the connexin-subtype in different ocular cell types.

Indeed, the permeability of HCs to reduced glutathione has been directly demonstrated, with the lens circulation system providing internal fibroblast cells with pathways for essential nutrients, removing potentially toxic metabolites and maintaining resting membrane potential [133]. It has been proposed that maintaining intercellular communication within the lens is crucial for supporting fibroblast function [134]. Cataract formation in humans and mice is caused by mutations in the GJ proteins of the lens fiber cells (LFCs) Cx46 and Cx50 [46]. Multiple studies have established an association between cataract formation and elevated intraocular calcium concentrations [135]. Research indicates that increased calcium levels in LECs lead to an increase in protease Lp82, which cleaves cytoplasmic proteins (lens proteins) [136]. Aggregation of these cleaved proteins scatters light, ultimately contributing to cataract development [137]. GJ channels and HCs are crucial for lens homeostasis, function, and transparency (Figure 2).

## 4. Treatment Methods

There are four main pharmacological inhibition approaches: molecular drug therapy, gap junction modulators, hemichannel blockers, and gene therapy. Among these, hemichannel blockers represent a key focus in drug research and development, potentially offering outstanding therapeutic potential [138].

### 4.1. Gap Junction Modulators

Carbenoxolone-modulating synaptic vesicle proteins can counteract inflammation-induced GJIC injury and astrocytic decoupling [139]. Retinoic acid modulates GJ permeability between horizontal cells of the mammalian retina [140].

### 4.2. Hemichannel Blockers

Therapeutic agents include mimetic peptides, nucleotides, and antibodies. HC blockers include mimetic peptide 5, Gap19, Gap26, Gap27, Tonabersat, and αCT1 [141]. The HC blocker Peptide5 inhibits inflammation and the inflammasome, while HC blockade prevents the development of clinical DR symptoms, representing a potential upstream therapeutic approach for DR. Tonabersat inhibits HC-mediated ATP release during both ischemia and reperfusion phases, with direct HC blockade confirmed electrophysiologically [142]. In clinical trials for migraine, the daily therapeutic dose of tonabersat was maintained between 20 and 80 milligrams [143]. This medication has also been proposed for the treatment of epilepsy [144]. Increased HC opening correlates with inflammasome pathway activation and inflammation, encompassing diverse pathologies, including ocular diseases such as AMD and DR [42]. By blocking the assembly of the inflammasome by Cx43, retinal inflammation is alleviated, thereby restoring the function of RPE cells and vascular integrity [145].

### 4.3. Gene Therapy

Gene therapy intervenes at the root cause of Cx-related diseases by regulating the expression of Cx genes or repairing mutated genes, making it suitable for hereditary or chronic progressive ocular conditions. In glaucoma models, the high-fidelity *CRISPR-Cas13 (hfCas13X)* system selectively targets and knocks down Cx43 expression in microglia. This mechanism enhances RGC survival and preserves optic nerve integrity, thereby improving visual function [146]. Silencing biglycan protein expression led to reduced retinal neovascularization in retinal pathological angiogenesis in mice [147]. Channel blockers exhibit high specificity, low toxicity, and strong targeting properties yet possess a short half-life requiring repeated administration such as intravitreal injections. They are suitable for acute inflammation and precise targeting of specific Cx subtypes. Regarding small-molecule drugs, advantages include convenient administration and prolonged half-life, though specificity is relatively low. They are suitable for chronic conditions and localized inflammation. In gene therapy, advantages encompass curative potential and long-term efficacy, though technical complexity and stringent safety validation are required. This approach is appropriate for hereditary Cx mutation disorders and chronic progressive diseases.

## 5. Conclusions and Future Perspectives

This review systematically elucidates the pivotal role of the Cx family in regulating hypoxic ocular diseases, with mechanisms centered on the sequence of events: hypoxia- altered channel function—ocular pathology. Cx subunit expression exhibits cellular specificity and disease association. Cx43, as a commonly regulated subtype, is extensively implicated in multiple pathologies, including diabetic retinopathy, high-altitude retinopathy, and glaucoma. It mediates damage through abnormal expression in critical cells such as RPEs, astrocytes, and RGCs. Cx36 focuses on neural signal transmission, with its downregulation being a causative factor in RGC dysfunction within glaucoma. Cx46 and Cx50 specifically regulate normal lens homeostasis and are directly implicated in the development of age-related cataracts. The pathological effects of Cxs are primarily realized through dual channels: GJs and HCs. Hypoxia-induced disruption of GJ communication impairs intercellular nutrient exchange and signal synchronization, while abnormal opening of HCs releases toxic molecules such as ATP and inflammatory factors. Collectively, these processes trigger ocular inflammation, disruption of the BRB, neuronal apoptosis, and pathological neovascularization. The Cxs-mediated cascade reaction ‘hypoxia -channel dysfunction-tissue injury’ serves as a crucial bridge linking the pathological mechanisms of hypoxic eye disease to therapeutic targets, providing a clear molecular target for overcoming current clinical treatment limitations. Despite the above research advances, the therapeutic impasse in hypoxic ocular diseases stems from a clear understanding of the pathological mechanisms, yet a limited number of intervention targets. A future challenge lies in the expression of multiple Cx subtypes across different ocular cell types; the use of drugs lacking subtypes or tissue selectivity may lead to dysfunction in normal intercellular communication. However, HC drugs have now entered preclinical research and hold considerable potential for novel therapeutic approaches. Future research will build upon existing findings to develop effective targeted therapies based on specific channel complex inhibitors, thereby providing innovative concepts and therapeutic strategies for clinical treatment.

## Figures and Tables

**Table 1:** Etiology of hypoxic ocular diseases and cellular localization of Cx.

References	Disease	Etiology	Connexin Localization	Subtype
[41]	Diabetic retinopathy	Retinal hypoxia, inflammation, endothelial dysfunction	RECs, pericytes, Müller cells, and RPE cells	Cx43
[42]	Age-related macular degeneration	Oxidative stress and hypoxia in the RPEs	Nerve fiber layer and RGCs	Cx43
[43]	High-altitude retinopathy	Retinal vascular dilation and tortuosity, cotton-wool spots	RECs, glial cells, and RGCs	Cx43
[28]	Ischemic retinopathy	Alterations in metabolic substrate availability, and mitochondrial dysfunction	Astrocytes	Cx43, Cx36
[44]	Glaucoma	Degeneration of RGCs and their axons	Glial cells and RGCs	Cx43, Cx36
[45]	Retinopathy of Prematurity	Pathological neovascularization	RECs	Cx43
[45]	Corneal hypoxic disease	Disturbances in corneal epithelial homeostasis, impairment of the corneal epithelial barrier function	Corneal epithelial cells	Cx43, Cx50, Cx26, Cx30
[46]	Age-related cataracts	Endoplasmic reticulum stress, reactive oxygen species (ROS) generation, and apoptosis persisting	Lens epithelial cell, fiber cells	Cx46, Cx50

## Data Availability

No new data were created or analyzed in this study. Data sharing is not applicable to this article.
